# Panniculectomy as a surgical option for the management of a deep surgical site infection after C-section in a morbidly obese woman: a case report

**DOI:** 10.1186/s13037-023-00363-y

**Published:** 2023-06-05

**Authors:** Joël Igor Kamla, Georges Motto Bwelle, Joel Noutakdie Tochie, Landry Wakheu Tchuenkam, Brigitte Wandji, Trevor Kamto, Agnès Esiéné

**Affiliations:** 1 Department of Surgery, Sangmelima Reference Hospital, Sangmelima, Cameroon; 2 Faculty of Medicine and Pharmaceuticals Sciences, University of Ebolowa, Ebolowa, Cameroon; 3 Faculty of Medicine and Biomedical Sciences, University of Yaounde I, Yaounde, Cameroon; 4 Digestive Surgery unit, Yaounde Central Hospital, Yaounde, Cameroon; 5 Department of Emergency Medicine, Anesthesiology and Critical Care, Douala Laquintinie Hospital, Douala, Cameroon; 6 Department of Surgery, Adlucem Hospital, Douala, Cameroon; 7 Obstetrics and Gynaecology Unit, Yaounde Central Hospital, Yaounde, Cameroon; 8 Department of Emergency Medicine, Anesthesiology and Critical Care, Yaounde Central Hospital, Yaounde, Cameroon

**Keywords:** Morbid obesity, Caesarean section, Surgical site infection, Panniculectomy

## Abstract

**Background:**

Obesity is an independent risk factor for the occurrence of surgical site infections (SSIs) following all types of surgeries, especially after Caesarean section (C-section). SSIs increase postoperative morbidity, health economic cost and their management is quiet complex with no universal therapeutic consensus. Herein, we report a challenging case of a deep SSI after C-section in a central morbidly obese woman managed successfully by panniculectomy.

**Case presentation:**

A 30-year-old black African pregnant woman with marked abdominal panniculus extending to the pubic area, a waist circumference = 162 cm and BMI = 47.7 kg/m^2^ underwent an emergency CS indicated for acute fetal distress. By day five post-operation, she developed a deep parietal incisional infection unremitting to antibiotic therapy, wound dressings and beside wound debridement till the 26th postoperative day. A large abdomen panniculus and maceration of the wound enhanced by central obesity increased the risk of failure of spontaneous closure; thus, an abdominoplasty by panniculectomy was indicated. The patient underwent panniculectomy on the 26th day after the initial surgery and her post-operative course was uneventful. Wound esthetics was satisfactory three months later. Adjuvant dietary and psychological management were associated.

**Conclusion:**

Post-Caesarean deep SSI is a frequent complication in obese patients. A panniculectomy may be a safe and promising therapeutic surgical option with good cosmetic results and little postoperative complications when used in a multidisciplinary anti-obesogenic approach.

**Supplementary Information:**

The online version contains supplementary material available at 10.1186/s13037-023-00363-y.

## Background

Obesity refers to an excess of adipose tissue defined clinically by a body mass index (BMI) ≥ 30 kg / m^2^ [1]. Obesity is an emerging major public health concern worldwide which is fueled by the "epidemiological transition of diseases" characterized by a global increase in urbanization and westernization of cultures predominant in low-and middle-income areas like Sub-Saharan Africa [2]. It is worth to mention that approximately one-third of the general population is either overweight or obese and that all age groups regardless of gender are affected by obesity [2]. The marked abnormal accumulation of fat in morbidly obese persons leads to ill-health sequelae in several body systems like the endocrinologic, cardiovascular, respiratory, neurological, osteoarticular and gastrointestinal systems as well as psychological and abdominal wall complications [3]. Obesity is also an independent factor of mortality and a decrease in life expectancy has been correlated with the severity of obesity [3]. Given that pregnancy is physiologically associated with a gradual weight gain eventually lost during the postpartum period, obesity in pregnancy is defined on the basis of the pregestational BMI[1]. A BMI ≥ 40 kg / m^2^ is conventionally termed morbid obesity [4]. In addition to the aforementioned obesity-related complications[3], obesity in pregnancy leads to significant perinatal complications in the antepartum, intra-partum and postpartum periods [1, 4]. Antepartum complications are spontaneous miscarriages, gestational diabetes mellitus, hypertensive disorders of pregnancy, and preterm labour [1, 5]. During labour, obesity is associated with an increment in the risk of induction of labour, prolonged labour and C-sections. [1, 5]. Postpartum, surgical site infections, venous thromboembolism, and postpartum depression are more common [1, 5]. More specifically with respect to surgical site infections (SSIs), perinatal obesity doubles the likelihood of SSIs [6]. The incidence of SSIs after C-section in obese women varies between 3 and 24% and their management is quiet challenging and complex [6–8]. In Cameroon, a Sub-Saharan African dubbed “Africa miniature”, the prevalence of obesity in the adult population is estimated at 15.1% [9]; in pregnancy, it is estimated at 16% [10]. Hence, obesity is a frequent health problem seen during pregnancy follow-ups and pregnant women undergoing surgery with myriad of complications amongst which, SSIs. Herein, we report a challenging case of a deep incisional SSI following C-section in a central morbidly obese woman managed by panniculectomy.

## Case presentation

A 30-year-old black sub-Saharan African woman, G_1_P_0_, at 42 weeks of gestation with an uneventful past history was admitted to our maternity unit for labour-like pains of six hours duration. She had a good pregnancy follow-up with no pregnancy pathology.

Clinical findings were not ill-looking, normal vital signs, a waist circumference of 162 cm, a weight of 124 Kg, a height of 1.61 m, a BMI of 47.7 kg/m^2^ with marked abdominal panniculus extending to the pubic area (grade III panniculus). Fetal heart rates were normal during uterine contractions and she had a clinically adequate pelvis. On vaginal examination, the cervix was dilated at 2 cm, membranes were intact, and the fetus was in the cephalic presentation. We concluded on a latent phase of labour in a central morbidly obese post-term primiparous woman. Twelve hours later, the fetus presented with severe bradycardia during uterine contractions which warranted the indication of an emergency C-section for acute fetal distress.

**Fig. 1:**
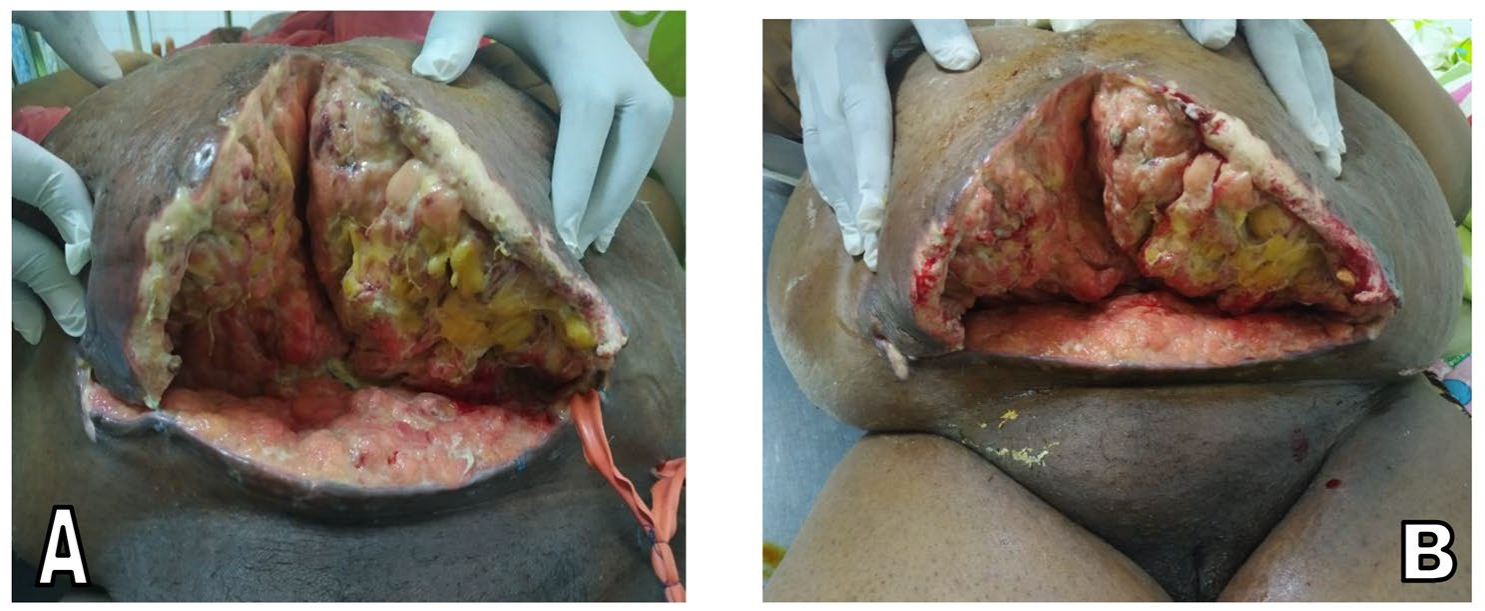
Aspect of wound after removal of stitches **A**: Wound on day 5 following debridement **B**: Good wound detersion on day 16

After obtaining the patient’s informed written consent, an anesthetic assessment graded her ASA III and an emergency C-section was performed under general anesthesia and orotracheal intubation. Antibiotic prophylaxis with ceftriaxone 2 g intravenous was started 30 min before surgical incision. The initial surgical access was a Pfannestiel incision secondarily extended with a median sub-umbilical incision due to difficulties in surgical access and dissections. A life vigorous crying male newborn weighing 4000 g was extracted, followed by placenta delivery, hysterorarrphy and skin closure. Antibiotic prophylaxis was continued in the post-operative period. On post-operative day five, suppuration was noticed at the operative wound. This was first managed by daily wound dressings and de-escalation to a second broad-spectrum antibiotic in conformity with microbiologic susceptibility test results of the wound suppuration. Due to the persistence of the purulent discharge on day 26 post-operation, stitches were removed, revealing a deep incisional SSI with areas of necrosis (Fig. 1.A). Progressive debridement associated with dressings was done for two weeks. Once the wound became clean and had granulated (Fig. 1.B), the surgical team staffed for a secondary wound closure versus an abdominoplasty. Given the risk of failure due to wound maceration maintained by central obesity (current waist circumference at 152 cm) with a large panniculus extending to the pubis, an abdominoplasty, more precisely a panniculectomy was indicated, followed by nutritional and dietary management.

**Fig. 2:**
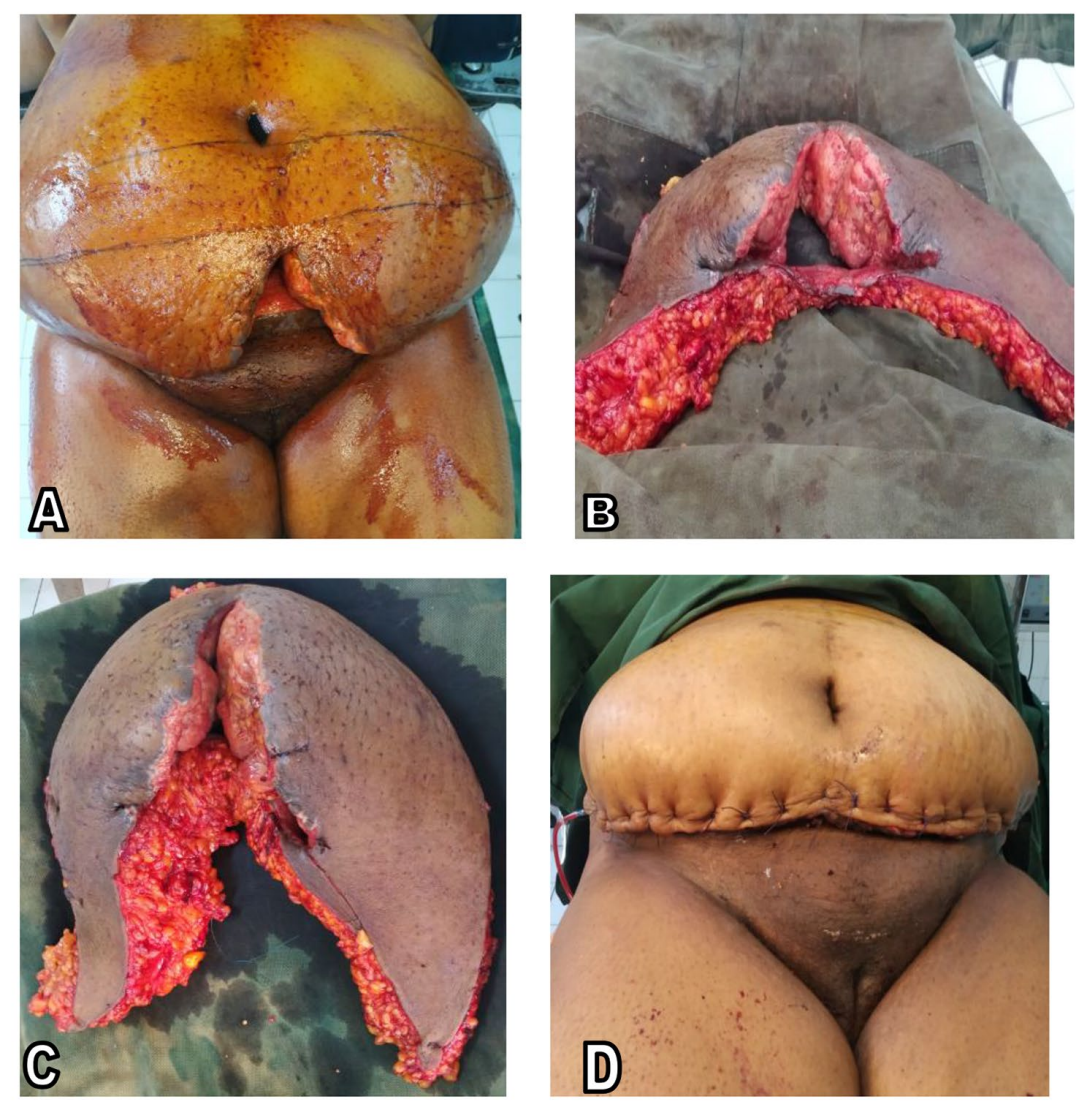
Panniculectomy **A**. Marking of the upper limit of the abdominal panniculus to be resected. **B.** Aspects of the superior and inferior margins of the incision. **C**. Skin and fatty tissue resected weighing 3700 Kg.

The patient was re-operated on day 26 following the initial C-section under epidural anesthesia. The surgical procedure performed was as follows (see Fig. 2): (i) tracing the incision’s lower and upper margins (see Fig. 2.A). The lower margin was located 8 cm above the commissure of the vulva and extended laterally and superiorly towards the antero-superior iliac spines; removing the initial Pfannestiel incision. The upper margin was an arciform lower umbilicus line with lower concavity, from one antero-superior iliac spine to the other. After checking on bimanual palpation that the suture would be tension-free, a modification of the upper limit was made; (ii) following marking of the incision, the second stage was the incision proper, which was done on the tracing aforementioned, giving an orange quarter incision; (iii) the next step was subcutaneous dissection of the aponeurotic fascia of the broad muscles of the abdominal wall, followed by resection of the sub-umbilical cutaneous-adipose plane. Then succeeded the detachment of the upper flap with rigorous hemostasis ensured, tension-free lowering of the flap down to the lower supra-pubic bank; (iv) finally, an approximation of the banks of the flap and skin suturing was done in three planes (deep adipose tissues, subcutaneous tissues and skin), then two lateral parietal suction drains were placed.

**Fig. 3:**
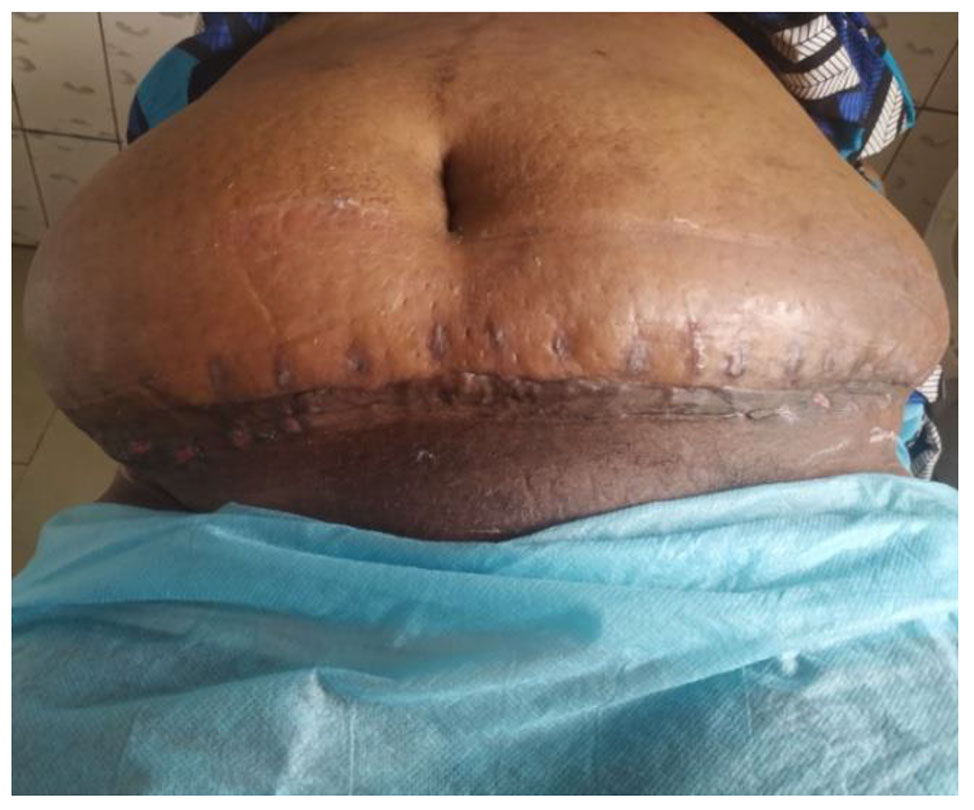
Aspect of the scar at post-operative day 90

An Enhanced Recovery After Surgery protocol was initiated. The post-operative course was uneventful on analgesics, antibiotics and venous thromboembolic disease prophylaxis. The drains were removed on post-operative day 5 and the patient was discharged seven days after the panniculectomy. A seroma was observed on post-operative day 8 and resolved completely a few days later after simple wound dressings. The appearance of the scar as well as that of the sub-umbilical skin was satisfactory on post-operative day 90 (Fig. 3). The patient had psychologist and nutritionist consults. A low-caloric diet was prescribed to prevent similar complications during future pregnancies and/or surgeries.

## Discussion

Obesity is a chronic metabolic disorder increasingly encountered in children, adolescents, adults, and pregnant women [11]. It is an independent risk factor for the occurrence of SSIs following many types of surgical interventions [12, 13], particularly, following C-section. [13]. There exist alterations in the vascularisation of the subcutaneous adipose tissues, which in turn alters the normal healing process and enhances the occurence of seromas and hematomas after C-section and later on SSIs [14]. Finally, obesity especially at the level of the folds is responsible for a maceration that maintains the infection. Once there is an infection at the surgical site, its management meets the general principles of all wound infection management : wound debridement, cleansing, dressing and coverage (reconstruction) [15]. The optimization of the treatment of SSI also involves the management of risk factors which in our case is mainly maceration. The initial incision was a Pfannestiel, performed at the lower abdominal fold, an area where maceration is very high, given the grade III panniculus [16]. Therefore, limiting maceration in this area could be achieved by reducing the abdominal panniculus.

Panniculectomy is an integral part of plastic surgery procedures. It is increasingly practiced given the rise in incidence of obesity and bariatric surgery [17]. In this case, it corrects the silhouette or contours of the abdomen by resection of excess abdominal skin and subcutaneous tissues formed as a result of excessive weight loss [16, 18, 19]. Apart from the aesthetic aspect responsible for most of its indications, panniculectomy can be performed for medical reasons [20]. It is indicated in cases of an important panniculus (grade 3 or overhanging panniculus) and symptomatic [20], that is to say associated with a severe skin infection or persistent ulceration despite conventional treatment. It may be the first step of a digestive or gynecological abdominal surgery requiring a sufficient operative exposure or to facilitate access to the abdominal cavity [16, 21]. In our case, it was carried out as part of the management of a persistent and sustained SSI by maceration induced by the excessive adipose panniculus.

Abdominal panniculectomy is a technique of transverse abdominoplasty. This is a low transverse anterior lipectomy; the principle is a resection of the abdominal panniculus by an incision in an orange quarter under the umbilicus associated with a lowering of the upper abdominal flap [16, 17]. Transposition of the umbilicus can be carried out depending on the case [17–19]. During this procedure, a plasty of the fascia or muscles of the abdominal wall is not performed, which makes it possible to differentiate panniculectomy from other abdominoplasty procedures. Thus, some authors refer to panniculectomy as a miniabdominoplasty [22].

The operative technique of panniculectomy described in this case has several variants [18]. The elements to be taken into account are: the xipho-umbilical distance, the umbilico-pubic distance, the presence of an umbilical hernia, an incisional hernia, an associated diastasis recti, the level of skin distension and the elasticity of the supra-umbilical region [23]. In the case of our patient, besides the huge adipose panniculus, the upper flap was not very elastic. This condition limited the extent of the area to be resected with a risk of having sutures under tension. Our patient had not undergone any previous bariatric surgery nor liposuction, and therefore, did not have an adequate melting of the adipose tissue sufficient to obtain a relaxation of tissue. As such, a simultaneous liposuction could have been performed intraoperatively. However, the infectious context was a contraindication for the latter.

The most common postoperative complications of panniculectomy are [17–19]: hematomas, seromas, SSIs, flap necrosis (in case of sutures under tension), an inaesthetic scarring (due to the ascension of the pubic flap in case of an excessive traction on the tissues) and rarely, thromboembolic complications seen in 0.56% of panniculectomy cases [16]. In the above case presented, we note the occurrence of a seroma which resolved a few days later with simple bandages.

Prevention of deep infection of the surgical site in morbidly obese patients during gynecological interventions by laparotomy would involve performing a panniculectomy at the same time [24, 25]. This additional procedure does not significantly increase operative time or blood loss. However, it is more easily conceived in elective surgeries, whereas our patient’s first surgical intervention was an emergency to safe her foetus.

Standard management of morbidly obese surgical patients like ours entails a multidisplinary approach by all the specialties involved in the management of obesity [26, 27]. These generally includes the following measures; dietary control, regular physical exercise, pharmacological treatment, psychological support and bariatric surgery [26, 27]. Bariatric surgery is indicated in the absence of sufficient weight loss despite a well-conducted multidisciplinary medical management for 6 - 12 months [26, 27]. Other eligibility criteria for bariatric surgery are: patient age between 18 to 60 years; BMI ≥ 40 kg/m2 or ≥ 35 kg/m2 with at least one comorbidity or BMI > 30 kg/m2 with diabetes mellitus or metabolic syndrome that is difficult to control [26, 27]. Furthermore, the patient must be well informed in advance and should accept long-term medical and post-surgical follow-up. Surgery should be done after multidisciplinary evaluation, in a patient with an acceptable surgical risk [24, 25]. Overall, our setting being a surgical infrastructural environment with limited equipment and skills for bariatric surgery, panniculectomy seems to be a promising safe practical adapted efficacious therapeutic option with minimal postoperative complications and satisfactory cosmetic outcomes when used in a multidisciplinary anti-obesogenic approach.

## Conclusion

The management of a deep incisional SSI in a central morbidly obese patient is complex and challenging especially in low- and middle-income countries with limited surgical infrastructure. Overall, this case highlights that panniculectomy could be a safe and promising surgical technique to reduce postoperative complications, achieve good postoperative esthetic results and palliate to the lack of bariatric surgery in low-and middle-income settings in the surgical management of obesity. This would, however, need further exploration in well-powered large-sample prospective studies.

## Electronic supplementary material

Below is the link to the electronic supplementary material.

Supplementary Material 1

## Data Availability

Data sharing is not applicable to this article as no data sets were generated or analyzed during the current study.

## List of Abbreviations

BMI: Body Mass Index
C-section: Cesarean section
Kg: Kilogram
m2: Square meter
SSI: Surgical site infection
