# Supplementary material for: Panniculectomy as a surgical option for the management of a deep surgical site infection after C-section in a morbidly obese woman: a case report
Source: Patient Saf Surg. 2023 Jun 5;17:14. doi: 10.1186/s13037-023-00363-y (PMC10242779; doi:10.1186/s13037-023-00363-y)
Supplement: Supplementary file 1 — Supplementary Material 1 [file 13037_2023_363_MOESM1_ESM.docx]

IGOR JOEL KAMLA
Faculty of Medicine and Pharmaceuticals Sciences
Tel: +237 656057208.

PO BOX: 886 Ebolowa. Cameroon

**kamlaigor@yahoo.fr**

ORCID ID: 0000-0001-7450-1025

Editor-in-chef

**Patient Safety in Surgery**

March 08, 2023

**Dear Editor,**

We are pleased to submit a new manuscript entitled “**Panniculectomy in the management of a deep surgical site infection in a morbidly obese woman following a cesarean section: a case report and mini review**.” for consideration by the journal Case Reports in Women’s health.

Our work reveals that a subumbilical panniculectomy may be necessary for the management of an infection of the surgical site after cesarean section, especially in the presence of a significant abdominal panniculus. Thus, we humbly hope to contribute to the improvement of maternal health in the world in general and in developing countries in particular.

We confirm that this work is original and has not been published elsewhere nor is it currently under consideration for publication elsewhere. All authors have read and approved the final version of the manuscript, and we have no conflicts to disclose.

Thank you for your consideration.

Sincerely,

Igor Joël Kamla

1. **Author names: Igor J Kamla^1,2*^;** **George M Bwelle^3,4^_;_ Joel N Tochie^3^; Landry W Tchuenkam^6^; Brigitte Wandji^7^; Trevor Kamto^3^; Agnès Esiéné^3,8^.**
2. **Full title of article: Panniculectomy in the management of a deep surgical site infection in a morbidly obese woman following a cesarean section: a case report and mini review.**
3. **list of authors with affiliations:**

| Full names and email addresses of all co­authors | | | |
| --- | --- | --- | --- |
| First name | **Middle name** | **Last name** | **email** |
| Igor | Joël | Kamla | [kamlaigor@yahoo.fr](mailto:kamlaigor@yahoo.fr) |
| Georges | Motto | Bwelle | [gbwelle@gmail.com](mailto:gbwelle@gmail.com) |
| Joel | Noutakdie | Tochie | [joeltochie@gmail.com](mailto:joeltochie@gmail.com) |
| Landry | Wakheu | Tchuenkam | [landrytchuenkus@gmail.com](mailto:landrytchuenkus@gmail.com) |
| Brigitte |  | Wandji | wandji.brigitte@gmail.com |
| Trevor |  | Kamto | trevorkamto@yahoo.com |
| Agnès |  | Esiéné | esieneagnes@hotmail.com |

4. **corresponding author**

- **name:** Igor Joel Kamla
- **Email:** **kamlaigor@yahoo.fr**
- **Affiliation:** Faculty of Medicine and Pharmaceuticals Sciences of the University of Ebolowa, Cameroon
- **Orcid ID:** 0000-0001-7450-1025

5. **number of attached files:**

- number of figures: 3
- cover letter: 1
